# Technologies for Arsenic Removal from Water: Current Status and Future Perspectives

**DOI:** 10.3390/ijerph13010062

**Published:** 2015-12-22

**Authors:** Nina Ricci Nicomel, Karen Leus, Karel Folens, Pascal Van Der Voort, Gijs Du Laing

**Affiliations:** 1 Laboratory of Analytical Chemistry and Applied Ecochemistry, Ghent University, Coupure Links 653, B-9000 Gent, Belgium; nrpnicomel@yahoo.com (N.R.N.); Karel.Folens@UGent.be (K.F.); 2 Department of Inorganic and Physical Chemistry, Center for Ordered Materials, Organometallics and Catalysis (COMOC), Ghent University, Krijgslaan 281-S3, B-9000 Gent, Belgium; Karen.Leus@UGent.be (K.L.); Pascal.VanDerVoort@UGent.be (P.V.); 3 Department of Engineering Science, College of Engineering and Agro-Industrial Technology, University of the Philippines Los Baños, 4031 Laguna, Philippines

**Keywords:** arsenic, pollution, water purification technology, engineered nanoparticles, adsorption, metal organic framework

## Abstract

This review paper presents an overview of the available technologies used nowadays for the removal of arsenic species from water. Conventionally applied techniques to remove arsenic species include oxidation, coagulation-flocculation, and membrane techniques. Besides, progress has recently been made on the utility of various nanoparticles for the remediation of contaminated water. A critical analysis of the most widely investigated nanoparticles is presented and promising future research on novel porous materials, such as metal organic frameworks, is suggested.

## 1. Introduction

Being the 20th most abundant trace element in the earth’s crust, arsenic (As) is a constituent in approximately 245 mineral species, which are predominantly ores containing sulfide, copper, nickel, lead, cobalt, or other metals [1,2]. Arsenic is widely used in various fields such as electronics, agriculture, wood preservation, metallurgy, and medicine [3]. These anthropogenic sources contribute to the release of arsenic to the environment in addition to its release from natural geological sources, for instance, by weathering of arsenic-containing rocks and volcanic activities.

Arsenic is a naturally occurring metalloid that is very mobile in the environment. Its mobility largely depends on the parent mineral form, oxidation state, and mobilization mechanisms [4]. In terms of oxidation state, arsenic can exist in four forms, which are arsenite (As(III)), arsenate (As(V)), arsenic (As(0)), and arsine (As(III)). Among these four arsenic species, the most prevalent forms, which are commonly found in water, are the inorganic arsenite and arsenate [5]. 

**Figure 1:**
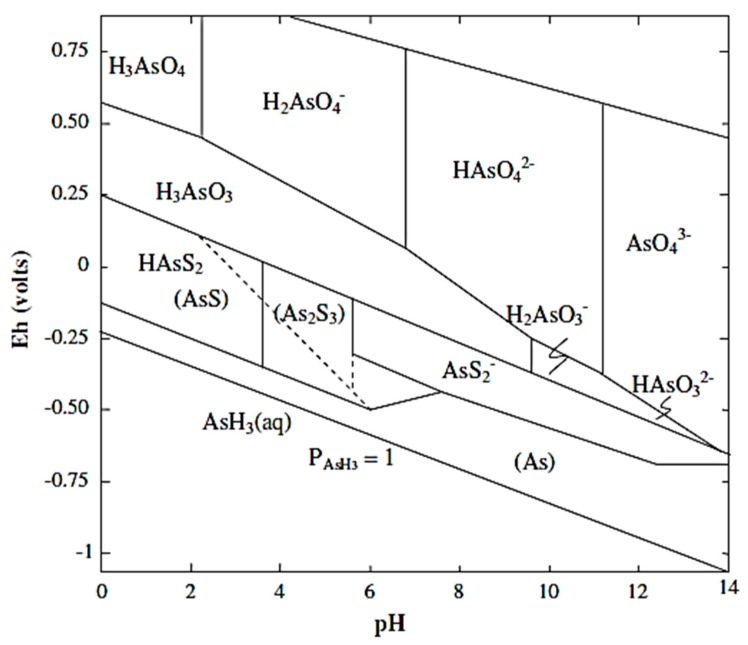
Eh-pH diagram for arsenic at 25 °C and 101.3 kPa (Adapted with permission from [17]). Arsenite and arsenate are the main species expected in environmentally relevant solutions. More oxic conditions, e.g., surface waters, stimulate the formation of arsenate. Moreover, the threshold potential required to form arsenate is lowered at high pH.

Because of slow redox transformations, arsenite and arsenate are present in both reduced and oxidized environments [4]. However, under anoxic reducing conditions (e.g., subsurface waters, reduced sediments), arsenic primarily exists as arsenite, whereas arsenate is prevalent in aerobic oxidizing environments, such as surface waters [6]. The pH also plays an important role in determining the state of arsenic [7]. Figure 1 shows the Eh-pH diagram of arsenic species in the system As-O_2_-H_2_O at a temperature of 25 ºC and total pressure of 101.3 kPa. Given a particular pH and redox potential, the speciation of arsenic, including its oxidation state, can be determined through this diagram [8]. This information is particularly useful in the determination of arsenic toxicity, taking the fact that the different arsenic oxidation states possess different toxicities into account [9]. Moreover, considering the fact that negatively charged arsenate (*i.e.*, H_2_AsO_4_^−^ and HAsO_4_^2−^) is generally much easier to remove compared to uncharged arsenite (*i.e.*, H_3_AsO_3_), this Eh-pH diagram can assist in the selection of optimum environmental conditions for arsenic removal [10].

Arsenic is known to be highly toxic to all life forms [11]. This element has been classified by the World Health Organization as a group 1 human carcinogenic substance [12]. Recently, many studies have been conducted regarding the environmental fate and behavior of arsenic due to several arsenic pollution cases worldwide and the hazards associated with these. Upon chronic intake of inorganic arsenic being present in concentrations above 50 μg/L in drinking water, different kinds of skin lesions (e.g., hyperpigmentation, hyperkeratosis) and cancers (e.g., skin, lung, kidney, bladder) can develop, which are collectively termed as arsenicosis [11]. In literature, it is well reported that inorganic arsenic species are more toxic than the organic species mono-methylarsenate (MMA) and dimethylarsinate (DMA). The toxicity of these species increases in the order: DMA–MMA–arsenate–arsenite [13]. As(III) is more harmful for human health than As(V) as it is more cytotoxic, genotoxic, mobile, and soluble [5,11,14]. With the accumulation of trivalent intermediates in the human body, there is a higher possibility of developing arsenic-induced diseases [15,16]. 

Exposure to arsenic can be through ingestion of arsenic-contaminated water or food and contact with arsenic-contaminated air. Reports have shown that elevated levels of arsenic in drinking water primarily contribute to human arsenic toxicity in the world [14,18,19]. Based on World Health Organization (WHO) guidelines, arsenic concentrations in drinking water should be strictly limited to 10 μg/L [6]. As groundwater is generally the main source of drinking water, levels exceeding this standard can often be linked to the contamination of groundwater by geothermal processes, mineral dissolution (e.g., pyrite oxidation), mining activities, desorption in oxidizing environments, and reductive desorption and dissolution [20]. In regions where contaminated drinking water is not the main source of arsenic for inhabitants, intake of food grown in areas with elevated arsenic concentrations in soils and irrigation water represents the primary cause of arsenic toxicity [11,21]. 

In 2012, it was estimated that about 202 million people worldwide are exposed to arsenic concentrations in drinking water above 50 μg/L [18]. Comparing this to an estimate of 130 million people in 2001, it can be inferred that there was a substantial increase in the number of people affected [12]. 

Worldwide, groundwater arsenic contamination is worst in Asian countries, especially in Bangladesh and West Bengal, India [22,23]. In both areas, the majority of the population depends on tube wells for water supply. Since the arsenic source is geogenic in nature, it has been reported that 79.9 million and 42.7 million people in Bangladesh and India, respectively, are exposed to contaminated groundwater having concentrations above 50 µg/L [19,24]. In Bangladesh, the arsenic concentration in some tube wells is as high as 4730 µg/L [22].

For the past three decades, several studies have shown that drinking arsenic-contaminated water should be one of the major concerns for the health of mankind [25,26]. Thus, strategies to avoid arsenic contamination of the groundwater and/or to alleviate the impact of such contamination need to be developed in an attempt to reduce the health risks associated with the intake of arsenic-contaminated water. In the following, a comprehensive overview is presented of the conventional techniques used for the removal of As species from water. Moreover, besides the use of nanoparticles for the treatment of arsenic-contaminated water, some novel porous adsorbents will be presented in this work which could act as superior adsorbent materials in the near future due to their outstanding characteristics, e.g., high pore volume and surface area. 

## 2. Conventional Techniques for Removal of as from Water

**Figure 2:**
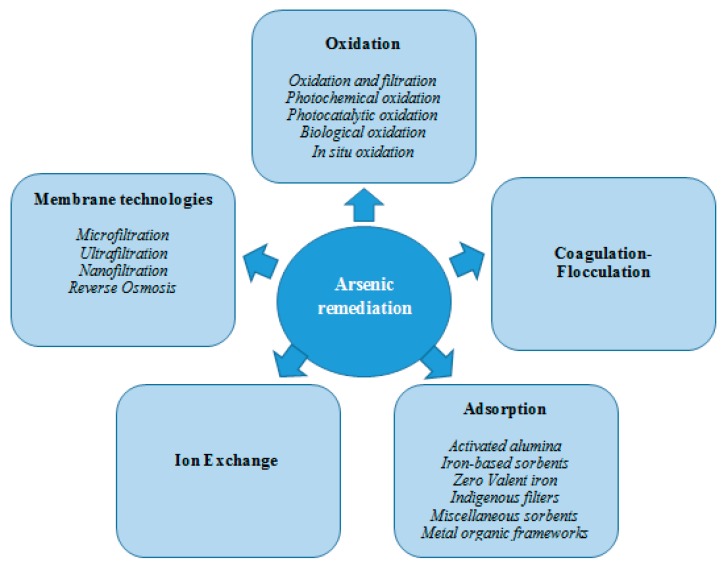
Various techniques used for the removal of arsenic from water.

The chemistry and composition of arsenic-contaminated water are the major factors determining the removal of arsenic [11]. Most of the available removal technologies are more efficient for arsenate given that arsenite is predominantly non-charged at pH below 9.2 [27]. This makes the trivalent form of arsenic less available for precipitation, adsorption, or ion exchange. Accordingly, treatment technologies are believed to be more effective by using a two-step approach consisting of an initial oxidation from arsenite to arsenate followed by a technique for the removal of arsenate [5]. Figure 2 summarizes the presently available technologies that can be used for the removal of arsenic from water.

### 2.1. Arsenic Removal by Oxidation Techniques

Oxidation involves the conversion of soluble arsenite to arsenate. This alone does not remove arsenic from the solution, thus, a removal technique, such as adsorption, coagulation, or ion exchange, must follow [27]. For anoxic groundwater, oxidation is an important step since arsenite is the prevalent form of arsenic at near neutral pH [11]. Aside from atmospheric oxygen, many chemicals, as well as bacteria, have already been used to directly oxidize arsenite in water and these are enumerated in Table 1.

**Table 1:** Different oxidants used to oxidize arsenite to arsenate, their operating conditions, properties, and efficiencies.

Oxidants	Operating pH	Initial as Concentration	Type of Water	Remarks	Reference
Oxygen and ozone	7.6–8.5	46–62 µg/L	Groundwater	Oxidation of As(III) by ozone is faster than by pure oxygen or air. In less than 20 minutes, complete oxidation was obtained using ozone, whereas five days were needed to oxidize 57% and 54% of As(III) using pure oxygen and air, respectively.	[28]
Chlorine	8.3	300 µg/L	Deionized water	As(III) was completely oxidized to As(V) by active chlorine when its initial concentration was greater than 300 µg/L. Stoichiometric rate was 0.99 mg Cl_2_/mg As(III).	[29]
Chlorine dioxide	8.12	50 µg/L	Groundwater	After one hour contact time, 86% oxidation yield was achieved. This relatively high value is mainly due to the presence of some metals in water that could assist the catalysis of As(III) oxidation.	[30]
Monochloroamine	8.12	50 µg/L	Groundwater	Very long contact time is needed to obtain effective As(III) oxidation. An oxidation yield of only 60% was achieved after 18 h.	[30]
Hypochlorite	7	500 µg/L	Groundwater	Given a hypochlorite concentration of 500 µg/L, there was a complete oxidation of As(III) to As(V).	[31]
Hydrogen peroxide	7.5–10.3	50 µg/L	Freshwater and seawater	The efficiency of As(III) oxidation improved as pH was increased from 7.5 to 10.3	[32]
Potassium permanganate	8.12	50 µg/L	Groundwater	Oxidation was completed after one minute.	[30]
*Photocatalytic oxidation* (UV/H_2_O_2_)	8	100 µg/L	Groundwater	Combining hydrogen peroxide (H_2_O_2_) with ultraviolet (UV) radiation resulted in an efficient As(III) oxidation. As UV dose increases, oxidation efficiency also increases. 85% of As(III) was oxidized to As(V) at a UV dose of 2000 mJ/cm^2^.	[33]
*Biological Oxidation* (e.g., chemoautotrophic arsenite-oxidizing bacteria (CAOs))	-	-	-	CAOs can participate in the oxidation of arsenite to arsenate through the use of oxygen (or nitrate) as terminal electron acceptors during the fixation of inorganic carbon into cell material.	[34]
*In situ oxidation*	-	-	Groundwater	Oxygenated water is pumped into the groundwater aquifer to reduce As concentrations to <10 µg/L.	[35]

In developing countries, atmospheric oxygen, hypochlorite, and permanganate are the most commonly used oxidants. Oxidation of arsenite with oxygen is a very slow process, which can take hours or weeks to complete [36]. On the other hand, chemicals, such as chlorine, ozone, and permanganate, can rapidly oxidize As(III) to As(V) as presented in Table 1. However, despite this enhanced oxidation, interfering substances present in water need to be considered in selecting the proper oxidant as these substances can greatly affect and dictate the kinetics of As(III) oxidation [11]. For instance, the oxidation rate of arsenite by ozone can be greatly reduced if S^2−^ and TOC are present in water [37]. Also, in another study, it was shown that competing anions and organic matter in groundwater greatly affect the use of UV/titanium dioxide (TiO_2_) in arsenic oxidation [38]. Furthermore, this involves a complex treatment, which produces an As-bearing residue that is difficult to dispose. Thus, to efficiently remove arsenic from a solution by oxidation, oxidants should be selected carefully. Moreover, all cited disadvantages of oxidation alone make it a less competent method for arsenic removal.

### 2.2. Coagulation-Flocculation 

Coagulation and flocculation are among the most employed and documented techniques for arsenic removal from water [27,39]. In coagulation, positively charged coagulants (e.g., aluminum sulphate (Al_2_(SO_4_)_3_), ferric chloride (FeCl_3_)) reduce the negative charge of colloids, thereby making the particles collide and get larger. Flocculation, on the other hand, involves the addition of an anionic flocculant that causes bridging or charge neutralization between the formed larger particles leading to the formation of flocs. During these processes, dissolved arsenic is transformed by the chemicals into an insoluble solid, which undergoes precipitation later [40]. Alternatively, soluble arsenic species can be incorporated into a metal hydroxide phase and be co-precipitated [27]. Either way, solids can be removed afterwards through sedimentation and/or filtration. 

Arsenic removal efficiency of different coagulants varies as a function of pH. Below pH 7.6, Al_2_(SO_4_)_3_, and FeCl_3_ are equally effective in removing arsenic from water [41]. Between the two inorganic arsenic species, most researchers suggested that arsenate is more efficiently removed compared to arsenite and that FeCl_3_ is a better coagulant than Al_2_(SO_4_)_3_ at pH higher than 7.6 [41,42,43,44,45]. However, despite their reported inferior performance compared to ferric chloride, aluminum-based coagulants were still able to reduce arsenic concentrations to below the maximum concentration level (MCL) of 10 μg/L given that the initial concentration is 280 µg/L [29]. Table 2 shows a list of the coagulants used in arsenic removal, together with their operating conditions, properties, and efficiencies. 

**Table 2:** Different coagulants used to remove arsenic, their operating conditions, properties, and efficiencies.

Coagulant	Operating pH	Initial as Concentration	Type of Water	Remarks	Reference
Ferric Chloride	7.0	2 mg/L	Distilled water	At an optimum FeCl_3_ dosage of 30 mg/L, As(III) and As(V) removal efficiencies were approximately 45% and 75%, respectively. Arsenic removal was enhanced at higher FeCl_3_ concentrations, however, residual iron after coagulation exceeded MCL of iron in drinking water.	[46]
Alum	7.0	20 µg/L	River water	About 90% of initial As(V) concentration was removed from the solution using 40 mg/L Al_2_(SO_4_)_3_ ·18 H_2_O. As(III) removal with alum was negligible even at higher alum doses.	[47]
Zirconium(IV) Chloride	7.5	50 µg/L	Distilled water	The percentage removal of As(V) with 2 mg/L ZrCl_4_ dosage was approximately 55%. This value increased at pH 6.5 and decreased at pH 8.5. In contrast to that of As(V), the removal efficiency of As(III) was approximately 8% regardless of pH.	[48]
Titanium(III) Chloride	7.5	50 µg/L	Distilled water	With 2 mg/L TiCl_3_, As(III) and As(V) removal efficiencies of 32% and 75% were achieved, respectively. Both As(III) and As(V) removal were highly pH dependent.	[48]
Titanium(IV) Chloride	7.5	50 µg/L	Distilled water	As(V) removal was highly pH dependent, whereas As(III) removal was independent of pH. With 2 mg/L TiCl_4_ dosage, approximately 55% of As(V) was removed, while As(III) removal was 26%.	[48]
Titanium(IV) Oxychloride	7.5	50 µg/L	Distilled water	Both As(V) and As(III) removal were pH dependent. The percent removal of As(V) with 2 mg/L TiOCl_2_ dosage was 37%. Given the same conditions, As(III) removal was about 20%.	[48]
Zirconium(IV) Oxychloride	7.5	50 µg/L	Distilled water	With 2 mg/L ZrOCl_2_ dosage, approximately 8% and 59% of As(III) and As(V) were removed, respectively. As(V) removal was highly pH dependent, whereas As(III) removal was independent of pH.	[48]
Ferric Sulfate	7.0	1 mg/L	Double distilled water	As(III) removal efficiency of 80% was achieved with 25 mg/L Fe_2_(SO_4_)_3_ dosage.	[49]
Titanium(IV) Sulfate	7.0	1 mg/L	Double distilled water	Ti(SO_4_)_2_ was employed for enhanced As(III) removal. The removal efficiency of As(III) was 90% at a coagulant dose of 25 mg/L.	[49]

The major drawback of coagulation-flocculation is the production of high amounts of arsenic-concentrated sludge [11]. The management of this sludge is necessary so as to prevent the consequence of secondary pollution of the environment. Moreover, treatment of sludge is costly. These limitations make this process less feasible, especially in field conditions [40].

### 2.3. Membrane Technologies 

In view of drinking water production, membrane filtration is a technique that can be used for the removal of arsenic and other contaminants from water. Typically, membranes are synthetic materials with billions of pores acting as selective barriers, which do not allow some constituents of the water to pass through [50]. A driving force, such as pressure difference between the feed and the permeate sides, is needed to transport the water through the membrane [51]. Generally, there are two categories of pressure-driven membrane filtrations: (i) low-pressure membrane processes, such as microfiltration (MF) and ultrafiltration (UF); and (ii) high-pressure membrane processes, such as reverse osmosis (RO) and nanofiltration (NF) [40,50]. The characteristics of these four processes are summarized in Table 3.

**Table 3:** Overview of pressure-driven membrane processes and their characteristics [51,52].

Parameter	Microfiltration (MF)	Ultrafiltration (UF)	Nanofiltration (NF)	Reverse Osmosis (RO)
Permeability (l/h.m^2^·bar)	> 1000	10–1000	1.5–30	0.05–1.5
Pressure (bar)	0.1–2	0.1–5	3–20	5–120
Pore size (nm)	100–10,000	2–100	0.5–2	< 0.5
Rejection Monovalent ions	−	−	−	+
Multivalent ions	−	−/+	+	+
Small organic compounds	−	−	−/+	+
Macromolecules	−	+	+	+
Particles	+	+	+	+
Separation mechanism	Sieving	Sieving	Sieving Charge effects	Solution-Diffusion
Applications	Clarification; Pretreatment; Sterilization	Removal of macromolecules, bacteria, viruses	Removal of organic compounds and some dissolved salts	Removal of salts

Using membranes with pore sizes between 0.1 and 10 µm, MF alone cannot be used to remove dissolved arsenic species from arsenic-contaminated water. Thus, the particle size of arsenic-bearing species must be increased prior to MF; the most popular processes for this being coagulation and flocculation [11]. In a study conducted by Han *et al.* [53], arsenic removal from drinking water was investigated through flocculation and MF wherein ferric chloride (FeCl_3_) and ferric sulphate (Fe_2_(SO_4_)_3_) were used as flocculants. Results showed that flocculation before MF leads to effective arsenic binding onto the ferric complexes present and subsequent arsenic removal in the permeate. However, the pH of the water and the presence of other ions are major factors affecting the efficiency of this arsenic immobilization. This can be a disadvantage of this technique especially when dealing with arsenite removal as this arsenic form has a neutral charge in the pH range of 4–10 [50]. Since arsenate is negatively charged in this pH range, it can bind by surface complexation resulting in efficient arsenate removal. Thus, for this technique to be effective, complete oxidation of arsenite to arsenate is needed. 

In the same way as MF, UF alone is not an effective technique for the treatment of arsenic-contaminated water due to large membrane pores [54]. To make use of this technique in arsenic removal, surfactant-based separation processes such as micellar-enhanced ultrafiltration (MEUF) can be utilized [55,56]. For instance, adding cationic surfactant to contaminated drinking water at a concentration above the critical micelle concentration (CMC) of the water will lead to formation of micelles, which can bind to negatively charged arsenic species. In effect, there will be arsenic removal in the permeate as the surfactant aggregates are large enough to pass through the membrane pores. Several studies have already focused on arsenic removal using MEUF. In one such study, the arsenate removal efficiency of different cationic surfactants was investigated [57]. Among the tested surfactants, hexadecylpyridinium chloride (CPC) showed the highest removal efficiency, *i.e.*, 96%. However, arsenic removal was also reported to decrease with decreasing pH. Moreover, despite the effective removal of arsenic, the concentration of the surfactant in the effluent is so high that it needs to be further treated with powdered activated carbon (PAC) before being discharged to the environment. 

Both NF and RO are suitable for the removal from water of dissolved compounds with a molecular weight above 300 g/mol [51]. These techniques can significantly reduce the dissolved arsenic level in water given that the feed is free from suspended solids and that arsenic is preferably present as arsenate [58]. In a study conducted by Sato *et al.* [59], it was shown that the removal efficiency for As(V) exceeded 85% for all investigated NF membranes, while that of As(III) was far too low. This is supported by the findings of Uddin *et al.* [60], who indicated that without oxidation of arsenite to arsenate, NF cannot comply with the MCL of arsenic in water. The same is the case for RO, as shown in several studies [50,61].

Although technically not a membrane system, diatomaceous earth (DE) filtration is a process that works very similar to that of membrane filters [62]. DE is a chalky sedimentary material containing fossil-like skeletons of microscopic water plants known as diatoms [63]. The size of diatoms ranges from 5–100 micrometers and are characterized by a porous structure having small openings of about 0.1 micrometer in diameter. The combined effect of small pore sizes and high porosity makes DE one of the most effective filters used to remove small particles at high water filtration rates [62,63]. Furthermore, this type of filter is odorless, tasteless, and chemically inert making it safe for filtering drinking water. Misra and Lenz [64] developed a method for removing arsenic and heavy metals from water using precipitated mixed hydroxides followed by DE filtration. In one of the laboratory-scale tests performed, an initial arsenic concentration of 100 µg/L was reduced by 90% using a reagent dose of 1000 mg/L. However, several drawbacks should also be considered, such as the need for pH adjustment, reagents, and a long conditioning time [64].

### 2.4. Adsorption and Ion Exchange

Adsorption is a process that uses solids as medium for the removal of substances from gaseous or liquid solutions [11]. Basically, substances are separated from one phase followed by their accumulation at the surface of another. This process is driven mainly by van der Waals forces and electrostatic forces between the adsorbate molecules and the adsorbent surface atoms. This makes it important to characterize first the adsorbent surface properties (e.g., surface area, polarity) before being used for adsorption [39]. 

A wide variety of sorbents has already been studied in several research areas as shown in Table 4. These include activated carbon, coal, red mud, fly ash, chicken feathers, kaolinite, montmorillonite, goethite, zeolites, activated alumina, titanium dioxide, iron hydroxide, zero-valent iron, chitosan, and cation-exchange resins. The table illustrates that iron-based adsorption is an emerging technique for the treatment of arsenic-contaminated water. This can be explained by the fact that there exists a high affinity between inorganic arsenic species and iron [65]. Iron can remove arsenic from water either by acting as a sorbent, co-precipitant or contaminant-immobilizing agent, or by behaving as a reductant [40]. 

Adsorption has been reported as the most widely used technique for arsenic removal due to its several advantages including relatively high arsenic removal efficiencies [66,67], easy operation, and handling [68], cost-effectiveness [69], and no sludge production [11]. However, adsorption of arsenic strongly depends on the system’s concentration and pH. At low pH, arsenate adsorption is favored, whereas for arsenite, maximum adsorption can be obtained between pH 4 and 9 [70]. Moreover, contaminated water does not only contain arsenic; it is always accompanied by other ions, such as phosphate and silicate, competing for the adsorption sites [71]. Aside from the system’s conditions, the effectiveness of adsorption in arsenic removal can also be hindered by the type of adsorbent itself. As shown in Table 4, a number of adsorbents have already been studied for the removal of arsenic from water. However, most conventional adsorbents have irregular pore structures and low specific surface areas, leading to low adsorption capacities. Lack of selectivity, relatively weak interactions with metallic ions, and regeneration difficulties can also confine the ability of these sorbents in lowering arsenic concentrations to levels below MCL [72,73].

**Table 4:** Comparative evaluation of different sorptive media previously used for arsenic removal.

Adsorbent	Type of Water	Optimum pH	Adsorbent Dosage (g/L)	Surface Area (m^2^/g)	Temperature (°C)	Sorption Capacity (mg/g)		References
						As(III)	As(V)	
Coconut-shell carbon	Distilled water	5.0	5	1200	25	-	2.40	[74]
Coconut-shell carbon pretreated with Fe(III)	Distilled water	5.0	10	-	25	-	4.53	[74]
Coal-based carbon	Distilled water	5.0	5	1125	25	-	4.09	[74]
Copper-impregnated coconut husk carbon	Distilled water	6.5	2	206	30	20.35	-	[75]
Rice polish	Deionized double-distilled water	7.0	20	452	20	0.14	0.15	[76]
Sorghum biomass	Deionized water	5.0	10	-	-	3.6	-	[77]
Fly ash	Distilled water	4.0	1	0.8 *	20	-	30	[78]
Activated alumina	Drinking water	7.6	1–13	370	25	0.18	-	[66]
Modified chicken feathers	Synthetic water	4.0	10	-	20	0.13	-	[79]
Allyl alcohol-treated chicken feathers	Synthetic water	7.0	10	-	25	0.115	-	[80]
Eggshell membrane	Distilled water	7.0	8	-	30	-	24.2	[81,82]
Synthetic zeolite H-MFI-24	Deionized water	6.5	2	450	20	-	35.8	[83]
Granular titanium dioxide	Groundwater	7.0	1	250.7	13.4	32.4	41.4	[84]
Granular ferric hydroxide (GFH)	Deionized-distilled water	6.5	0.25	240–300	20	-	1.1	[85]
Iron oxide-coated cement	Double-distilled water	7.0	30	-	15	0.73	-	[86]
Iron oxide-coated sand	Distilled water	7.5	20	-	27	0.029	-	[87]
Iron-oxide-coated manganese sand (IOCMS)	Deionized water	7.0	5	9.18 *	25	2.216	5.452	[88]
Iron-modified activated carbon	Deionized-distilled water	7.6–8.0	0.1–20	723	20-23	38.8	51.3	[89]
Amorphous iron hydroxide	Deionized water	6.0–8.0	1.6	-	-	28.0	7.0	[70]
Zero-valent iron	Groundwater	10.0	5	1.8 *	25	-	1.92	[90]
Goethite	Deionized water	6.0–8.0	1.6	-	-	22.0	4.0	[70]
Fe_x_(OH)_y_-Montmorillonite	Deionized water	6.0–8.0	1.6	165	-	13.0	4.0	[70]
Ti_x_H_y_-Montmorillonite	Deionized water	6.0–8.0	1.6	249	-	13.0	3.0	[70]
Natural siderite	Tap water	7.31	2	-	20	1.04	0.52	[91]
Kaolinite	-	5.0	100	33 *	25	-	0.86	[92]
Modified calcined bauxite	Double-distilled water	7.0	5	-	30	-	1.566	[93]
Activated red mud	Distilled water	7.25/3.50	20	-	25	0.884	0.941	[94]
Chitosan resin	Deionized distilled water	6.0	2	-	40	4.45	-	[95]
Cerium-loaded cation exchange resin	Deionized water	5.0–6.0	10	-	25	2.5	1.03	[96]
Surface-modified diatomite	Artificial wastewater	7.0	-	50–55 *	25	-	8.0	[97]

* Brunauer, Emmett and Teller (BET) surface area.

## 3. Application of Nanoparticles for Removal of Arsenic from Water

Recently, advances in nanoscience and nanotechnology have paved the way to the development of various nanomaterials for the remediation of contaminated water [40]. Due to their high specific surface area, high reactivity, and high specificity, nanoparticles have been given considerable environmental attention as novel adsorbents of contaminants, such as heavy metals and arsenic, from aqueous solutions [98]. Carbon nanotubes and nanocomposites, titanium-based nanoparticles, iron-based nanoparticles, and other metal-based nanoparticles are among the most widely used and investigated nanoparticles for the treatment of arsenic-contaminated water [72,99,100,101]. Table 5 presents a summary of the comparative evaluation of some nano-adsorbents used for arsenic removal. 

### 3.1. Carbon Nanotubes (CNTs) 

CNTs have been reported to be effective in the adsorption of various organic chemicals and metal ions after treatment with oxidants [102,103,104]. In a study conducted by Choudhury *et al.* [105], As(III) adsorption efficiency of Multiwall CNTs was approximately 34.22% after 30 min, given an initial As(III) concentration of 542 µg/L and a sorbent concentration of 1 g/L. Furthermore, the results revealed that Multiwall CNTs are able to remove arsenic to safe limits, but only for a low initial arsenic concentration.

CNTs can also be functionalized in order to increase removal efficiency for metal ions. Velickovic *et al.* [106] synthesized CNTs functionalized with polyethylene glycol (PEG) for the removal of As(V) and other metal ions from wastewater. It has been shown that the adsorption of these metal ions on PEG-CNTs is strongly pH dependent. Moreover, for an initial concentration of 10 mg/L and pH equal to 4, the maximum adsorption capacity of As(V) on this functionalized CNT was found to be 13.0 mg/g.

However, in general, CNTs may not be a better alternative for activated carbon as all-encompassing adsorbents. Nevertheless, CNTs still show potential in some applications wherein only small amounts of materials are required, which implies less material cost. These applications include polishing steps to remove recalcitrant compounds or pre-concentration of trace organic contaminants for analytical purposes [100].

### 3.2. Titanium-Based Nanoparticles 

Pena *et al.* [107] evaluated the effectiveness of nanocrystalline titanium dioxide (TiO_2_) in arsenic removal and in photocatalytic oxidation of As(III). Adsorption of arsenite and arsenate by nanocrystalline TiO_2_ reached equilibrium within four hours, whereas with commercial nonporous TiO_2_ particles, it was already reached in an hour. Furthermore, higher adsorption capacity was obtained using nanocrystalline TiO_2_, which can be due to its higher specific surface area than the nonporous TiO_2_ particles. At an equilibrium arsenic concentration of 45 g/L, more than 80% of both arsenic species was adsorbed by this nano-adsorbent. In terms of oxidation, nanocrystalline TiO_2_ was also shown as an efficient photocatalyst considering that arsenite was completely converted to arsenate within 25 min in the presence of sunlight and dissolved oxygen.

**Table 5:** Comparative evaluation of various nano-adsorbents for arsenic removal.

Nano-Adsorbent	Properties		Operating pH	Adsorbent Dosage (mg/L)	Temperature (°C)	Sorption Capacity (mg/g)		References
	Average Particle Size (nm)	Surface Area (m^2^/g)				As(III)	As(V)	
Multiwall carbon nanotubes functionalized with polyethylene glycol (PEG-MWCNTs)	17.4	22.5	4.0	0.1	25	-	13.0	[93]
Hydrous titanium dioxide	4.8	312	7.0	500	25	83.0	-	[95]
Iron-doped TiO_2_	108.0	-	7.0	4000	-	-	20.4	[108]
Ti-loaded basic yttrium carbonate (Ti-BYC)	10.0–30.0	82.0	7.0	1000	25	-	348.5	[109]
α-Fe_2_O_3_ nanoparticles	5.0	162.0	7.0	100	25	95.0	47.0	[104]
γ-Fe_2_O_3_ nanoparticles	7.0–12.0	168.73	-	-	-	67.02	-	[110]
Fe_2_O_3_ nanoparticles	12.3	-	6.0	100	-	20.0	4.9	[111]
Magnetite nanoparticles	20.0	69.4	6.5	400	25	8.0	8.8	[105]
Fe_3_O_4_ nanoparticles	5.0	178.48	7.0	60	-	46.06	16.56	[112]
Ceria nanoparticles	6.6	86.85	-	5000	30	18.02 *		[107]
CeO_2_–CNT	-	189.0	7.0	25	-	-	81.9	[113]
Zirconium oxide nanoparticles	10.8	98.0	7.0	100	-	5.2	6.0	[108]
Zirconium oxide nanoparticles	-	327.1	7.0	100	25	83.0	32.4	[109]

* As(total).

Another titanium-based nano-adsorbent being used in arsenic removal is hydrous titanium dioxide nanoparticles (TiO_2_·x H_2_O). These offer the advantage of being effective adsorbents for As(III) without the need for oxidation to As(V) or any pH adjustment before and after the adsorption process [38]. Xu *et al.* [114] synthesized hydrous TiO_2_ nanoparticles, which were tested for As(III) removal from laboratory-prepared and natural water samples. With a maximum adsorption capacity of 83 mg/g at near neutral pH and 96 mg/g at pH 9, application of TiO_2_·x H_2_O proved to be an effective, low-cost, and single-step process for the treatment of arsenic-contaminated water. However, because of their size, dispersion of these nanoparticles into the environment is to be expected. Thus, granulation of these nanoparticles into micron-sized particles or loading onto very porous host materials is needed.

A recent study conducted by Lee *et al.* [109] showed an enhanced arsenate removal in aqueous solution using Ti-loaded basic yttrium carbonate (BYC). The maximum adsorption capacity of Ti-loaded BYC at pH 7 was reported to be 348.5 mg/g, which is 25% higher than either BYC or yttrium hydroxide. This can be attributed to its increased specific surface area (82 m^2^/g) and surface charge (PZC:8.4). Moreover, Ti-loaded BYC also displayed high adsorption capacities in a wider pH range (pH 3–11). This adsorbent also performed well even in the presence of coexisting anionic species (e.g., phosphate, silicate, bicarbonate). However, this study did not report on the potential of Ti-loaded BYC to adsorb arsenite. 

### 3.3. Iron-Based Nanoparticles 

**Figure 3:**
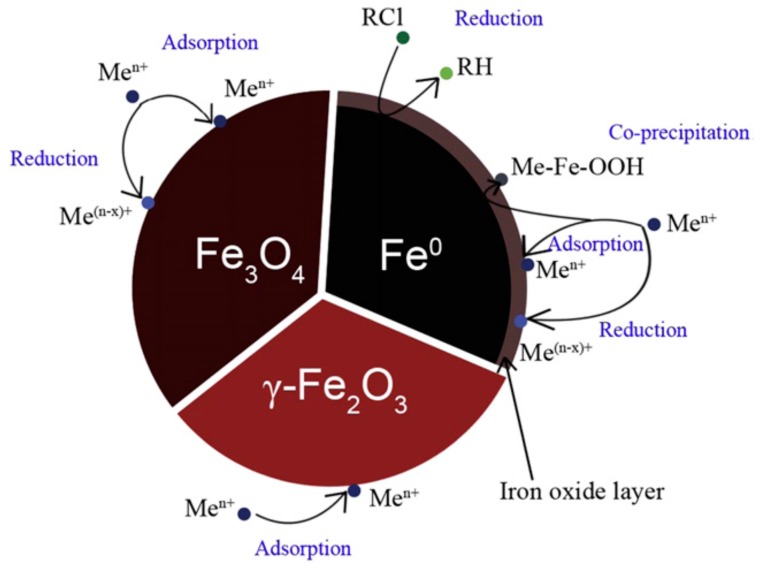
Schematic model of the removal mechanisms of nZVI, Fe_3_O_4_, and γ-Fe_2_O_3_ (Adapted with permission from [101]).

Among the most important nanomaterials studied for the treatment of arsenic-contaminated water are iron-based nanoparticles, which include zero-valent iron nanoparticles (nZVI) and iron oxide nanoparticles (*i.e.*, Fe_3_O_4_, and γ-Fe_2_O_3_). The oxidation state of iron in these particles has a major influence on their capability to remove contaminants [101]. Several mechanisms are involved in these removal processes (Figure 3).

#### 3.3.1. Zero-Valent Iron Nanoparticles (nZVI)

Several laboratory studies have demonstrated that the application of nZVI is an effective technology for transforming pollutants into their nontoxic form [115]. For instance, dyeing reagents can be adsorbed effectively to functionalized nZVI, which exhibited a maximum adsorption capacity of 191.5 mg/g for one type of dye studied [116]. In this case, adsorption was the result of donor-acceptor bonds formed in the reaction between the functional group –NH_2_ on the nZVI surface and the –OH group on the target compound. As for heavy metals, adsorption and co-precipitation are generally accepted mechanisms involved in removal by nZVI [72]. As schematically shown in Figure 3, these mechanisms occur because an iron oxide shell is formed once nZVI is brought in contact with air or water. Removal of arsenic is a widely studied example [117,118]. 

Using high resolution X-ray photoelectronic spectroscopy (HR-XPS), Ramos *et al.* [119] studied the arsenic immobilization mechanism using nZVI. Primarily due to the core-shell structure of nZVI, it was shown that both reductive and oxidative mechanisms take place upon application of nZVI. This structure is characterized by a highly reducing metal core and a thin layer of amorphous iron (oxy)hydroxide that helps in the coordination and oxidation of As(III). However, despite this reported advantage of nZVI being a versatile technology for arsenic remediation, other studies also indicated that this nano-adsorbent is disadvantageous when it comes to synthesis [120].

#### 3.3.2. Iron Oxide Nanoparticles

Iron oxide nanomaterials are increasingly becoming prevalent in the field of arsenic removal because of their ability to remove arsenic five to ten times more effectively than their micron-sized counterparts [72]. This enhanced uptake capacity for metals, in general, can be attributed to their high surface-to-volume ratios [121]. In addition to this, iron oxide nanoparticles possess magnetic properties, which allow them to be conveniently separated from aqueous solutions [122]. 

Tang *et al.* [123] synthesized ultrafine α-Fe_2_O_3_ nanoparticles using a solvent thermal process to treat laboratory-prepared and natural water samples contaminated by arsenic. Studying the kinetics revealed that As(III) and As(V) removal by α-Fe_2_O_3_ nanoparticles can be achieved very rapidly. Within the first 30 minutes of contact, around 74% of As(III) was already removed with an α-Fe_2_O_3_ loading of 0.04 g/L and an initial As(III) concentration of 0.115 mg/L. For a solution containing 0.095 mg/L of As(V), 100% removal was achieved when the α-Fe_2_O_3_ loading was only half of that used for As(III). The synthesized nanomaterial, with a BET specific surface area of 162 m^2^/g and an average particle diameter of 5.0 nm, showed high arsenic adsorption capacities at around neutral pH conditions. Adsorption capacities for As(III) and As(V) were determined to be 95 mg/g and 47 mg/g, respectively. Moreover, this study showed that the presence of Cl^−^, NO_3_^−^, and SO_4_^2−^ in the water has only a minimal negative effect on arsenic adsorption. Only HPO_4_^2−^ and SiO_3_^2−^ could lower the arsenic adsorption substantially, especially the adsorption of As(V), when the concentrations of these two anions were high. 

The performance of magnetite (Fe_3_O_4_) nanoparticles in treating arsenic-contaminated water was investigated by Chowdhury and Yanful [124]. This nano-adsorbent has a BET surface area of 69.4 m^2^/g and a mean particle diameter of 20 nm. Results showed that arsenic adsorption by Fe_3_O_4_ nanoparticles is largely dependent on the pH of the solution, contact time, initial arsenic concentration, PO_4_^3−^ concentration in the solution, and the adsorbent concentration. At an initial arsenic concentration of 2 mg/L, maximum adsorption of both arsenic species was achieved at pH 2. Arsenate adsorption decreased rapidly when pH was above 7, whereas for arsenite, adsorption was more or less constant in the pH range 2–9. Furthermore, maximum arsenic adsorption capacities were calculated to be 8.0 mg/g and 8.8 mg/g for As(III) and As(V), respectively, using the Langmuir isotherm. The effect of phosphate on arsenic removal was also studied and results showed that removal percentages decreased with increasing phosphate concentration. This result is in accordance with the study conducted by Roy *et al.* [105] wherein the presence of phosphate at a concentration of 0.5 mg/L decreased As(III) and As(V) removal percentages by 13% and 25%, respectively. 

Mayo *et al.* [125] also studied As(III) and As(V) removal using nanocrystalline magnetite particles. Their results confirmed that the size of the nanoparticles has a significant effect on their adsorption behavior. Adsorption capacities for both arsenic species increased about 200 times when the particle size was decreased from 300 nm to 12 nm. Hristovski *et al.* [98] investigated the removal of arsenate through batch experiments carried out using 16 commercially available nanoparticles of metal oxides. Most of these nanoparticles removed >90% of arsenate from almost all water matrices, with TiO_2_, Fe_2_O_3_, ZrO_2_, and NiO nanopowders performing best. These nanoparticles showed the highest removal efficiencies, exceeding 98%, except for ZrO_2_ in groundwater.

### 3.4. Other Metal-Based Nanoparticles 

#### 3.4.1. Ceria Nanoparticles

Feng *et al.* [126] conducted batch experiments to study the adsorption of arsenic on ceria nanoparticles. Results showed that arsenic removal by this nanomaterial is pH-dependent. For arsenate, adsorption increased when the pH increased from 1 to 6, and then decreased as pH continued to increase beyond 6. Similar trends were observed for arsenite, although arsenite adsorption was observed to continuously increase in the pH range from 1 to 8. Moreover, Langmuir adsorption isotherms revealed that the adsorption capacities of these nanoparticles were 17.08 mg/g, 18.02 mg/g, and 18.15 mg/g at 10, 30, and 50 °C, respectively, indicating that arsenic adsorption is favored at higher temperatures. 

#### 3.4.2. Zirconium Oxide Nanoparticles

Being chemically stable, non-toxic, and insoluble, zirconium-based oxides could also be an option for drinking water purification [127]. One of the few studies regarding this group of nanoparticles was conducted by Cui *et al.* [128]. They synthesized amorphous zirconium oxide (am-ZrO_2_) nanoparticles by a hydrothermal process for arsenic removal from water. Through kinetics studies, it was shown that by using only a relatively low dosage of am-ZrO_2_ nanoparticles (*i.e.*, 0.10 g/L), arsenic concentrations in water can be reduced to levels below MCL within 12 h for As(V) and 24 h for As(III). Moreover, the adsorption process was observed to be effective under near neutral pH conditions and does not need any pretreatment or post-treatment. Maximum adsorption capacities of these am-ZrO_2_ nanoparticles were found to be 83.2 mg/g and 32.5 mg/g for As(III) and As(V), respectively.

### 3.5. Disposal of Arsenic-Contaminated Nanoparticles

The nanoparticles may need to be disposed when their saturation capacity is reached. For other metals and organics, nanoparticles may be recovered through combustion [67]. However, in the case of arsenic-loaded materials, combustion may not be ideal as arsenic oxides are volatile and are easily released to the atmosphere during the combustion process, which creates a new environmental hazard [129]. Therefore, the most attractive option to handle arsenic-loaded nanoparticles currently seems to be encapsulation through stabilization-solidification, followed by secure landfill disposal [67,130,131]. The first step, stabilization-solidification, is a popular technique used to convert a potentially hazardous liquid or solid waste into a less or non-hazardous waste before it is disposed in secure landfills [131].

### 3.6. Regeneration and Reuse 

In cases where process economy dictates that immediate disposal is not cost-effective, regeneration of the adsorbent seems to be the preferred option. Several studies suggest that the maximum adsorption capacity of metal-based nanoparticle adsorbents remains almost constant after several cycles of regeneration and reuse [132,133,134,135]. Moreover, pH is considered as an important factor in the desorption of metals from the adsorbents. This is in accordance with the results obtained by Tuutijärvi *et al.* [132] concerning the desorption characteristics of arsenate and the recovery of the adsorbent maghemite (γ-Fe_2_O_3_) nanoparticles. Among the five alkaline solutions studied (*i.e.*, NaOH, Na_2_CO_3_, Na_2_HPO_4_, NaHCO_3_, and NaOAc), 0.1 M NaOH showed the highest desorption efficiency of 90%. Moreover, desorption was proven to be affected by pH and the concentration of the alkaline solution. By increasing the concentration of NaOH to 1 M, full desorption of arsenate was achieved. 

However, other authors also reported a reduced adsorption capacity after regeneration. Saiz *et al.* [129] aimed to analyze the regeneration and reusability of arsenate-loaded Fe_3_O_4_^@^SiO_2_. When comparing HCl and NaOH, the latter provided the better desorption performance at a concentration of 0.01 M. This alkaline condition was further used to evaluate the long-term performance of the regeneration process, wherein sorbent functionalization steps (*i.e.*, protonation of amino groups and coordination of Fe^2+^) were performed in between adsorption and desorption stages. After five adsorption-desorption cycles, the desorption yield decreased by 26%, while the re-adsorption yield only diminished by 5.7%. A decreasing trend of adsorption capacity was also reported by Deliyanni *et al.* [136] in a study regarding the sorption of As(V) ions by akaganéite-type nanocrystals. It was found that regeneration of adsorbent was not complete. Moreover, about 25%–30% of akaganéite’s capacity was lost in each regeneration step, which means that the adsorbent must be replaced after three or four regeneration steps. 

In some aforementioned cases, nanoparticles can be easily regenerated and reused for the removal of arsenic considering that their adsorption capacities are more or less constant even after several cycles of regeneration. This may be an advantage of using nanoparticles as adsorbents. For some nanomaterials not retaining their adsorption capacity during regeneration, this may not be a total disadvantage restricting their potential use as most reagents used in the uncomplicated preparation of these nano-adsorbents are cheap and readily available [129]. In addition, these materials have relatively high adsorption capacities that can outweigh the costs needed to replace the materials after several cycles.

### 3.7. Stability Issues 

Nanoparticles have been proven to be effective in the adsorption of heavy metals. However, since they are usually present as fine or ultrafine particles, nanoparticles have low energy barriers, causing them to aggregate and achieve a stabilized state [99,137]. Aggregation decreases the free surface area of the nanoparticles, thereby reducing their adsorption capacity and reactivity [101]. Moreover, the mobility of the particles decreases, which further contributes to reducing their effectiveness. To overcome the problems associated with aggregation, two solutions were reported in literature. A first solution is the impregnation of nanoparticles into porous materials or surface coatings [68,138]. Some of the widely used host substrates are activated carbon [139], bentonite [140], sand [141], alumina membranes [142], and ion-exchange resins [143]. As for surface coating, there have been reports mentioning that a thick layer of surface modifiers may reduce the reaction rate, although removal capacity was enhanced due to an increased number of active sites [101]. Thus, tradeoff between stability and reactivity must be studied well. The other solution is the design and synthesis of micronano hierarchically structured sorbents, which can balance high adsorption capacity and nanoparticle stability [144].

## 4. Metal Organic Frameworks as Novel Porous Adsorbents 

Metal organic frameworks (MOFs) are porous crystalline hybrid solids that are comprised of inorganic and organic building blocks connected to each other by coordination bonds [145,146]. In general, the inorganic components are metal ions or a cluster of metal ions, in which the most often used are the transitional ones, such as Fe^3+^, Zn^2+^, and Al^3+^. On the other hand, organic components, also known as linkers, are multidentate organic ligands, which can be electrically neutral, anionic, or cationic [147]. Carboxylates are the most widely used anionic linkers due to their ability to make metal ions aggregate, and thus, form more stable networks [148]. 

Because of their relatively simple and easy synthesis, high surface areas, tunable pore sizes and shape, coordinative unsaturated sites (CUS), and organic functionality, MOFs have gained significant attention in research and industry during the last two decades [145,149,150]. Moreover, these hybrid materials showed potential in various fields including hydrogen storage [151], gas adsorption [152,153], separation of chemicals [154], catalysis [155], drug delivery [156], magnetism [157], luminescence [158], and sensors [159]. 

Also, adsorption of hazardous substances from water can be one of the potential applications of MOFs, although their adsorption abilities have been less explored as compared to other materials, such as zeolites [160,161]. To some extent, this can be attributed to the fact that only a few classes of MOFs are stable in water for a longer time [162]. 

In comparison to nanoparticles, MOFs present two major advantages in adsorption applications. First is the presence of CUS or open metal sites in their structure that are readily accessible. Secondly, MOFs have high thermal and mechanical stability making them withstand aggregation problems that are very common in nanoparticles [144]. These advantages, together with their remarkably high porosities and high specific surface areas up to 10,450 m^2^/g, make MOFs perform better in removing heavy metals from water than other porous adsorbents [163,164,165]. 

Li *et al.* [166] studied the adsorption of arsenate from water and the characteristics of arsenate removal by MIL-53(Al). It was concluded that the adsorption rate was initially high, which enabled the MOF to reach 80% of its maximum adsorption capacity within 11 h. At pH 8, MIL-53(Al) reached a maximum removal capacity of 105.6 mg/g, which was observed to gradually decrease in strong acidic or alkaline conditions. In addition, a removal capacity of 15.4 mg/g was obtained at a lower equilibrium concentration of 10 μg/L. Through Fourier transform infrared spectroscopy (FT-IR) and X-ray photoelectron spectroscopy (XPS), it was determined that arsenate removal by MIL-53(Al) might be due to electrostatic adsorption and hydrogen bonds. A strong point of this MOF as an adsorbent is that its structure was maintained and no aluminum ions were detected in the water phase after the adsorption. This makes it a suitable material for drinking water treatment. However, the presence of competing anions can hinder the performance of MIL-53(Al) in arsenate adsorption. At a concentration of 1.9 mg/L, PO_4_^3−^ had the greatest impact as it was shown that MIL-53(Al) maintained only 13.5% of the maximum adsorption capacity (*i.e.*, 105.6 mg/g) with the presence of this competing anion. 

Zhu *et al.* [144] also investigated arsenate removal from aqueous solutions using iron and 1,3,5-benzenetricarboxylic MOF (Fe-BTC). This MOF showed relatively high arsenate adsorption capacity of 12.3 mg/g, which is about 2 times and 11 times higher than those of iron oxide (Fe_2_O_3_) nanoparticles and commercial iron oxide powders, respectively. Moreover, arsenate can be adsorbed by Fe-BTC in a wide pH range (*i.e.*, pH 2–12). Optimum removal efficiency was observed under acidic conditions. At pH levels above 12, removal efficiency dropped drastically as the MOF was being dissolved in strong basic conditions. It was also shown in this study that arsenic ions were adsorbed onto the interior of Fe-BTC and not on the outer surface. This explains why this MOF has a higher adsorption capacity compared to Fe_2_O_3_ nanoparticles since it provides more interior space. However, another MOF was reported to have better performance in arsenate removal from aqueous solutions [167]. In this study, MIL-53(Fe) was used with a maximum adsorption capacity of 21.27 mg/g, which is about two times more than that of Fe-BTC.

Zeolitic imidazolate framework-8 (ZIF-8) was also applied as a nano-adsorbent of arsenic species from water in a study conducted by Jian *et al.* [164]. Maximum adsorption capacities, obtained at a temperature of 25 °C and pH 7, were 49.49 and 60.03 mg/g for As(III) and As(V), respectively. ZIF-8 was only stable at neutral and basic conditions as high amounts of Zn^2+^ were detected in the water in acidic conditions, which decreased the efficiency of arsenic adsorption. Aside from this, the presence of competing anions, such as PO_4_^3−^ and CO_3_^2−^ can also negatively affect the adsorption of arsenic.

## 5. Conclusions and Perspectives 

Arsenic is recognized as a persistent contaminant in groundwater with severe impact on human health when exposed through, amongst other sources, drinking water. Arsenic emissions from natural sources, including not in the least certain Asian countries, and anthropogenic emissions urge for on-site remediation to reduce the toxicity risks. Conventional techniques generally focus on arsenate removal after an initial oxidation of arsenite by either atmospheric oxygen, bacterial activity, or chemical reagents. Increasing the particle size of soluble species is possible by a coagulation/flocculation process and allows removal by precipitation or membrane filtration in a consecutive step. Ion-exchange resins alternatively are capable of directly immobilizing As ions, but this process is subject to pH influences and competition from co-occurring ions such as phosphate or silicate. Nevertheless, practical use of these conventional and non-conventional techniques is still limited due to the fact that their adsorption capacities are still too low and there is a lack of potential to regenerate and reuse the adsorbents. 

Nanomaterials made of carbon, titanium, iron, ceria, or zirconium are an emerging class of adsorbents due to their high specific surface areas, high reactivity and high specificity. Nevertheless, the nanoparticles’ high surface energies mean they tend to aggregate in aqueous media, which results in a drastic decrease in surface area and therefore in a reduced capacity and selectivity, reducing the process lifetime and potential for real life application.

However, despite the number of studies conducted regarding nanoparticle stabilization for adsorption, little or no attention was given to a novel class of porous materials that was recently gaining considerable attention in other fields of research because of their outstanding properties. These are Metal Organic Frameworks (MOFs), which possess high surface areas, tunable pore sizes and shape, high thermal stability, and a relatively simple synthesis. Moreover, they have the advantages that they can be easily pre- or post-modified on the organic moieties for target specific compounds. MOFs now became subject of exploration for removing hazardous substances such as arsenic or fluoride from contaminated water streams. Their excellent adsorption capacities encourage further development of adsorption technologies towards reaching acceptable arsenic levels.
